# Masonry Columns Confined by Steel Fiber Composite Wraps

**DOI:** 10.3390/ma4010311

**Published:** 2011-01-21

**Authors:** Antonio Borri, Giulio Castori, Marco Corradi

**Affiliations:** Civil and Environmental Engineering Department, School of Engineering, University of Perugia, Via Duranti 93, 06125 Perugia, Italy; E-Mails: borri@unipg.it (A.B.); gcastori@strutture.unipg.it (G.C.)

**Keywords:** masonry column, steel fiber, mechanical testing, reinforcement, confinement

## Abstract

The application of steel fiber reinforced polymer (SRP) as a means of increasing the capacity of masonry columns is investigated in this study. The behavior of 23 solid-brick specimens that are externally wrapped by SRP sheets in low volumetric ratios is presented. The specimens are subjected to axial monotonic load until failure occurs. Two widely used types of masonry columns of differing square cross-sections were tested in compression (square and octagonal cross-sections). It is concluded that SRP-confined masonry behaves very much like fiber reinforced polymers (FRP)-confined masonry. Confinement increases both the load-carrying capacity and the deformability of masonry almost linearly with average confining stress. A comparative analysis between experimental and theoretical values computed in compliance with the Italian Council of Research (CNR) was also developed.

## 1. Introduction

For many years external confinement has been considered an effective method for strengthening reinforced concrete and masonry compression members due to the urgent need to upgrade deficient columns to meet current design standards. Recent catastrophic earthquake events in Southern Europe (Italy, Greece) and Asia Minor (Turkey, Iran, Iraq) have highlighted the vulnerable condition of old construction, which is typified by very low quality of masonry.

The subject of reinforcement of existing masonry structures is of notable importance in light of the world’s architectural heritage, and it is of particular interest in those areas struck by earthquakes, where the aim of reconstruction is a substantial seismic upgrading of existing constructions.

Steel jacketing has been extensively used to retrofit masonry columns and has proven to be very efficient in increasing the strength and ductility of the columns [1,2].

During the past decade efforts have been increasingly concentrated on the replacement of “traditional” steel reinforcement in masonry columns by FRP reinforcement. Most of the studies performed on FRP-jacketed columns in the reported literature concentrate on either experimental and/or analytical models. Many investigations have been conducted into the behavior of FRP-confined masonry and, as a result, a number of stress-strain models have been proposed. This kind of application has been of noticeable interest to designers, due to the fact that it is possible to reach increments of both load capacity and ductility. Many experimental tests and studies on the use of FRP as a strengthening material for masonry have confirmed the substantial properties of FRP materials. Krevaikas and Triantafillou [3], and Triantafillou [4], proposed a simple analytical confinement model to predict the response of FRP-confined masonry. Furthermore later studies by Corradi *et al.* [5], Aiello and Sciolti [6], Micelli *et al.* [7] and Di Ludovico *et al.* [8], have shown that the effectiveness of the wraps is dependent on the shape of the column and the stiffness of the FRP wraps. Due to the high anisotropy of FRP materials, square- and rectangular-section masonry columns were found to experience less increase in strength and ductility. Researchers have dedicated particular attention to the problem of the corners of columns with rectangular cross-sections, devising numerical procedures and formulae capable of predicting the confining action produced by the composites [9].

In the meantime, researchers and practitioners are looking for other innovative approaches to improve the retrofit of deteriorating masonry structures. One approach is by the use of high strength steel fiber, which offers ease of handling and speed of installation, as well as high strength-to-weight ratio.

There is a lack of knowledge on the stress-strain behavior of masonry columns confined with high strength steel cords. This new strengthening material, which can be made from coils found on the market, is available brass coated or galvanized with zinc, for greater protection against corrosion. The coils used here are about 30 cm wide and variable in length, and consist of a series of cords laid out parallel to each other, held together by a polyester mesh. This paper is concerned with the experimental study of masonry columns characterized by square and octagonal cross-sections confined with high strength steel fiber. The masonry specimens are wrapped with relatively low confinement volumetric ratios (λ = 0.31 ÷ 0.62%) so as to examine their confininng effect when SRP sheets are used as reinforcement in rehabilitation. Concerning its confining characteristics, SRP reinforcement exhibits a linear elastic behavior up to failure and exerts an ever-increasing confining pressure on the masonry core.

## 2. Test on Masonry Columns

### 2.1. Test Matrix

Twenty-three solid brick masonry columns were subjected to uni-axial compression in order to test the column strength confined by steel composites. Table 1 shows the experimental program and type of reinforcement. The purpose was to evaluate the increase in compressive strength of masonry columns produced by the reinforcement wrapping and to record the axial stress-strain curve. Another important objective was to find the failure mode of the masonry columns.

**Table 1:** Experimental program.

Specimen	Shape of Cross-section	Matrix Type	Reinforcement Type	Reinforcing Scheme
1	octagonal	-	-	-
2	square	-	-	-
3	square	epoxy	Type 1	Continuous wrap
4	square	epoxy	Type 1	Continuous wrap
5	octagonal	epoxy	Type 2	Continuous wrap
6	octagonal	epoxy	Type 2	Discontinuous wrap
7	octagonal	epoxy	Type 2	Continuous wrap
8	octagonal	epoxy	Type 2	Discontinuous wrap
9	octagonal	epoxy	Type 1	Continuous wrap
10	octagonal	epoxy	Type 1	Discontinuous wrap
11	octagonal	epoxy	Type 1	Continuous wrap
12	octagonal	epoxy	Type 2	Discontinuous wrap
13	octagonal	epoxy	Type 1	Discontinuous wrap
14	octagonal	epoxy	Type 1	Continuous wrap
15	octagonal	epoxy	Type 2	Discontinuous wrap
16	octagonal	epoxy	Type 2	Continuous wrap
17	square	epoxy	Type 1	Continuous wrap
18	square	epoxy	Type 1	Continuous wrap
19	square	epoxy	Type 1	Discontinuous wrap
20	square	epoxy	Type 1	Discontinuous wrap
21	square	epoxy	Type 1	Discontinuous wrap
22	square	epoxy	Type 1	Discontinuous wrap
23	square	epoxy	Type 1	Discontinuous wrap

**Figure 1:**
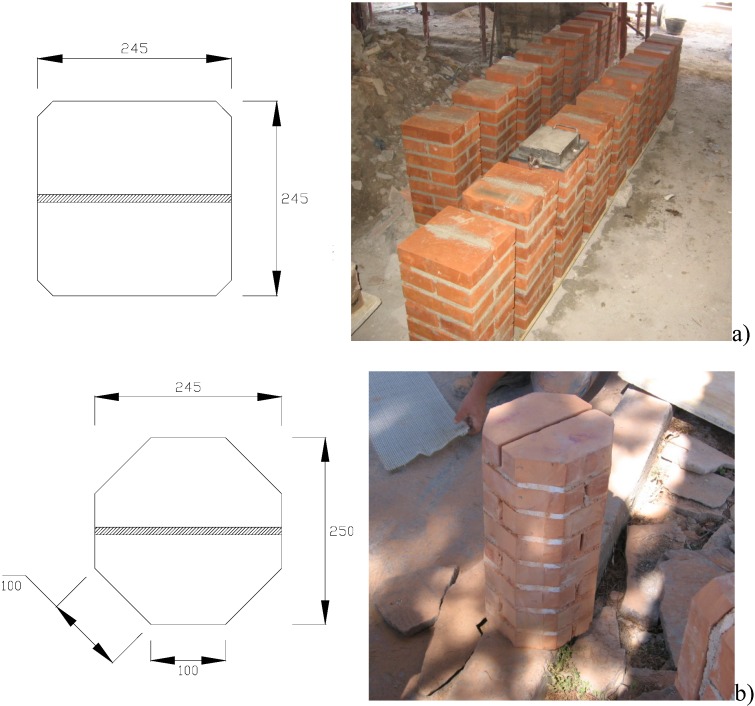
Geometry of cross-sections of masonry columns (dimensions in mm) **(a)** square cross-section; **(b)** octagonal cross-section.

Using 245 × 120 × 55 mm solid clay bricks, two types of columns of differing cross-sections (squares with sides of 245 mm and octagonals with sides of 100 mm) were constructed. Octagonal cross-section masonry columns are in fact quite common in Italy and the rest Europe in many historical constructions such as churches, monasteries and porticoes. Of the 23 samples in the series 10 had square cross-sections while the remaining 13 had octagonal sections. The geometry of the specimens with the two cross-sections is shown in Figure 1. All masonry columns were 500 mm tall, while the thickness of mortar bed joints was equal to 8 ÷ 10 mm.

Another test variable was the type of steel cords (Type 1 or 2). One distinctive feature of these materials is their macroscopic structure. All the fibers are made up of high-strength steel filaments covered with a layer of brass to prevent oxidation of the metallic cords. These cords are placed side by side and glued to a thin polyester mesh to allow them to be packaged in the form of a strip. Placing these metallic cords side-by-side and gluing them onto thin polyester meshes results in a product in the form of sheets with a density of 12 cords/inch (4.72 cords/cm), which are then wound on bobbins.

**Figure 2:**
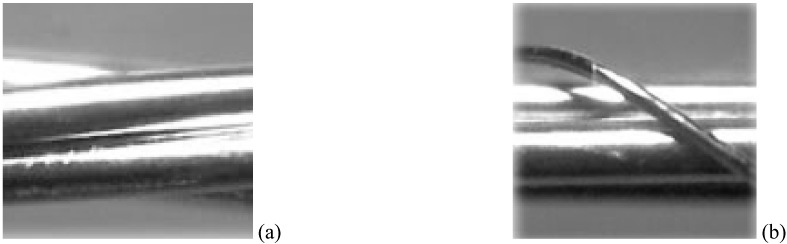
(**a**) 3X2 cord (Type 2); (**b**) 3SX cord (Type 1).

The Type 2 cord is made by twisting five individual filaments together (three straight filaments wrapped by two filaments at a high twist angle) (Figure 2a). The Type 1 cord results from winding four single high strength metallic filaments together: three filaments are wound together by a single external filament of a smaller diameter (Figure 2b). Type 1 was used only for reinforcement of square cross-section columns, and Type 2 for both square and octagonal cross-sections columns.

**Figure 3:**
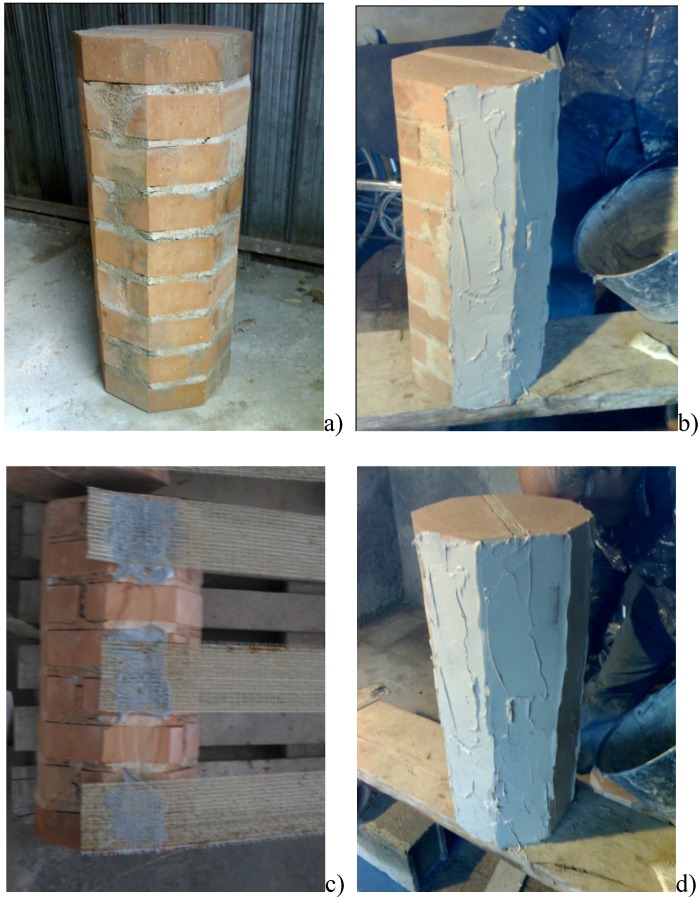
**(a)** Cleaning of the column surfaces; **(b)** Application of a first layer of matrix; **(c)** Application of a unidirectional SRP sheet; **(d)** Application of a second layer of epoxy resin or cementitious mortar.

The steel cords were glued by using a two-component epoxy resin. Reinforcement was executed in the following steps (Figure 3): (a) cleaning of the column surfaces of all extraneous material to improve the adhesion between resin and masonry; (b) application of a first layer of matrix; (c) application of a unidirectional SRP sheet; (d) application of a second layer of epoxy resin or cementitious mortar. All specimens were wrapped with orientation perpendicular to their axis.

**Figure 4:**
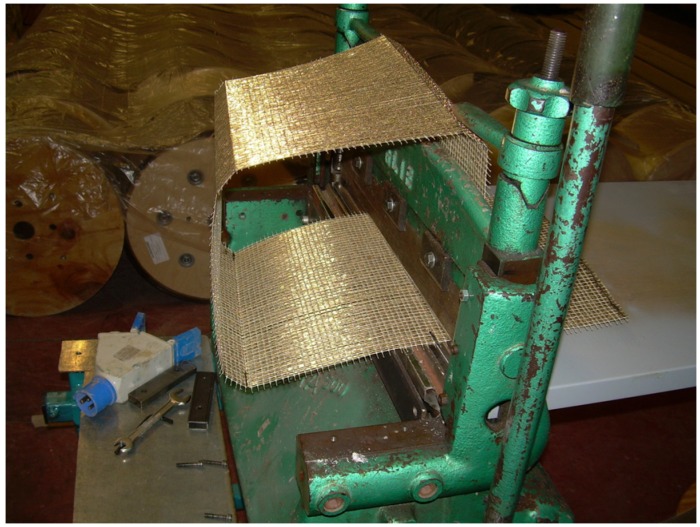
Cord cold bending (used only for square cross-section columns).

The steel fibers were cold-bended with an appropriate apparatus (Figure 4) to form a square section identical to the cross-sections of the masonry columns. This was necessary due to the bending stiffness of the steel filers which cause debonding of the fibers on the corners of the masonry columns.

Particular attention was given to surface preparation and corner reinforcement: Before wrapping the SRP sheets, masonry surface defects were filled with cementitious mortar. The edges of the columns were rounded with a radius of 30 mm to prevent any stress concentrations within the reinforcing layers. Wrapping of the SRP sheets took place after curing for at least 28 days in laboratory conditions.

**Figure 5:**
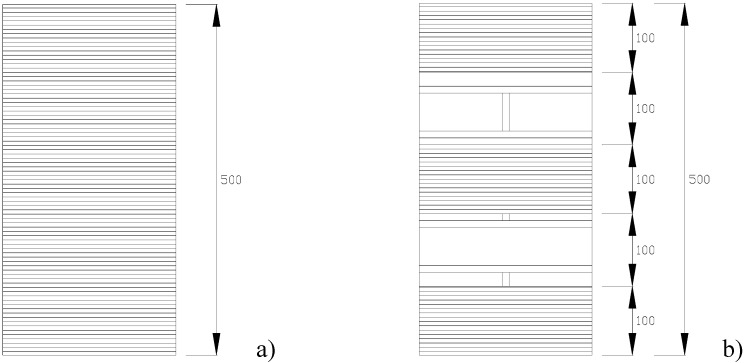
Reinforcement layout: **(a)** continuous wrap; **(b)** discontinuous wrap.

Two different configurations of the reinforcing system were investigated: SRP sheets are applied as external reinforcement along the perimeter of the masonry columns in the form of continuous and discontinuous wrap, respectively. To elaborate, in the first case the confinement was provided by a continuous laminate composed of two SRP strips of 300 mm width each (Figure 5a); whereas in the second case the confinement was provided by three SRP strips, 100 mm wide and 200 mm spaced (Figure 5b). In both cases, the overlap of the sheet in circumferential direction is 100 mm, and there is no lengthwise overlap.

The series of masonry columns are identified by a three index code, in which the first indicates the cross-section shape (S = square cross-section; O = Octagonal cross-section); the second indicates the type of steel cords (3SX = Type 1 cords; 3X2 = Type 2 cords); the third the reinforcing system (C = Continuous wrap; D = Discontinuous wrap).

### 2.2. Mechanical Characterization of Materials

Mechanical characterizations were performed for all materials used in the masonry columns (bricks and mortar) and in the reinforcement (fibers and epoxy-resins).

### 2.3. Cord Characterization

Tensile tests were performed on SRP laminates to determine the mechanical properties of the steel cords (Table 2) and to draw their stress-strain curves.

The mechanical properties of the metal cords were verified by tensile tests carried out on eight samples. Tests were executed using a 100 kN displacement-controlled universal testing machine. Steel fiber behavior turned out to be linear-elastic up to failure and experienced tensile failure. Average tensile strength and elastic modulus were equal respectively to 3311 MPa and 214560 MPa for Type 1, and 2511 MPa and 187,356 MPa for Type 2. The results substantially confirmed the values of the tensile strength given by the manufacturer on the technical sheet, with small variations (+3.5% and +4.7% for Type 1 and 2 respectively).

**Table 2:** Mechanical properties of the reinforcement provided by the manufacturer.

	Type 1	Type 2
Cord sectional area (mm^2^)	0.621	0.811
Tensile strength (MPa)	3199	2396
Elastic Modulus (MPa)	160,000	143,000
Ultimate strain (%)	1.55	1.16

### 2.4. Epoxy Resins

In accordance with ASTM D638 [10] and D695 [11] specifications, a mechanical characterization of the epoxy-resin was performed in order to find the tensile and compressive strength value, as well as the modulus of elasticity. Tests were performed on a Lloyd Instruments LR30K dynamometer using the controlling and measuring system R-Control Lloyd. Table 3 shows the test results of characterization of the epoxy putty used.

### 2.5. Bricks

The mechanical properties of the solid clay bricks—with dimensions 245 × 120 × 55 mm—used for masonry column construction were obtained by means of compression and bending tests, each of which was carried out on six samples. Uniaxial compression tests with a 245 × 120 mm cross-section gave a mean strength of 20.99 MPa, whereas the mean value of the bending tensile strength was 0.81 MPa. Compression tests were executed with the load direction orthogonal to the brick’s main dimensions.

**Table 3:** Results of traction and compression tests of epoxy-resin used.

Number of Samples	4
Tensile Strength (MPa)	25.21
Young Modulus E (MPa)	4510
Number of Samples	5
Compressive Strength (MPa)	65.54
Young Modulus E (MPa)	4634

### 2.6. Mortar

The mortar used was composed of Portland cement (10% in volume), sand (80% in volume) and hydraulic lime (10% in volume). The strength of the mortar was determined from bending and compression testing. Three 160 × 40 × 40 mm prisms were tested in flexure with three-point bending and six 40 × 40 × 40 mm cubes in compression. The 28-day average strength results were as follows: 3.56 MPa for flexion tests and 10.75 MPa for those of compression.

## 3. Test Setup

**Figure 6:**
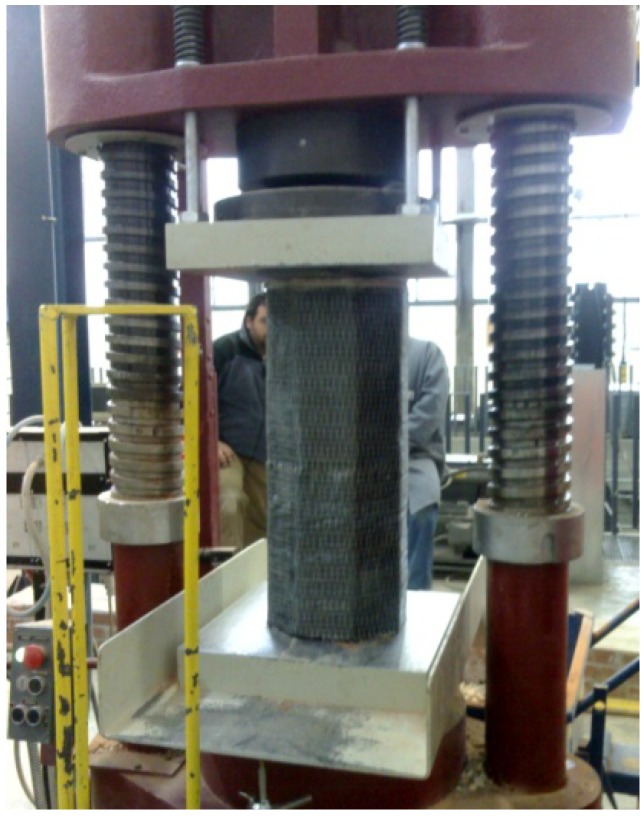
Test setup.

The tests were executed in load-control mode with a loading rate of 2/3 KN/s using a compression testing machine of 3000 kN capacity (Figure 6). The purpose was to evaluate the increase in compressive strength of masonry columns produced by the fiber wrapping and to record the axial stress-strain curve. Another important objective was to find the failure mode of the masonry columns. Loads were measured using a load cell and displacements were obtained using external linear variable differential transducers (LVDTs) mounted on the masonry columns (base length 500 mm). Loads and displacements were all recorded by a data acquisition system.

## 4. Experimental Results

### 4.1. Square Cross-Section Masonry Specimens

One of the ten specimens underwent testing without any type of reinforcement, to estimate strength and stiffness characteristics, while the remaining nine were confined with two different types of reinforcing system (continuous or discontinuous wrap) embedded in an epoxy matrix.

**Figure 7:**
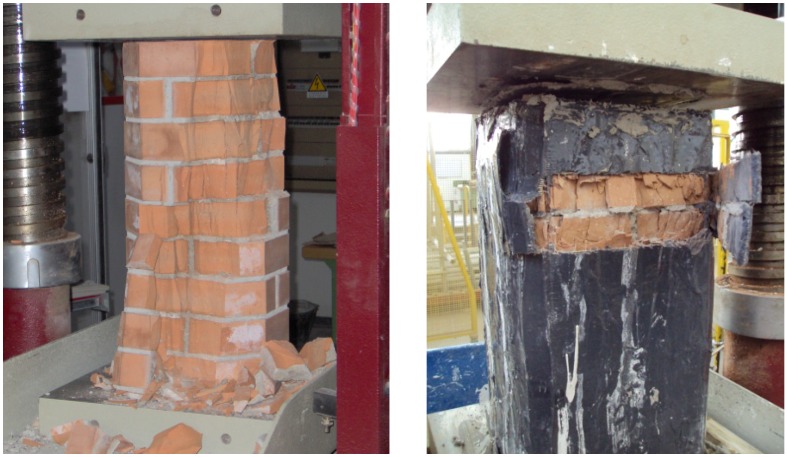
Failure modes of square cross-section column: **(a)** unreinforced specimens; **(b)** reinforced specimens.

The control specimen failed at a load of 708 kN, corresponding to a compressive strength of 11.91 MPa (Table 4). The compression stress caused the columns to fracture with vertical cracks formed through mortar joints and solid clay bricks (Figure 7a).

**Table 4:** Square cross-section masonry columns: Experimental results.

Series	Specimen	Volumetric ratio λ (%)	Average max compression load P (kN)	Average compressive strength (MPa)	Normalized strength P_confined_/P_unconfined_	Average load peak axial strain
Unconfined	2	-	708	11.91	1.00	0.007
S-3X2-C	3, 4, 17, 18	0.60	1510	26.65	2.24	0.0225
S-3X2-D	19, 20, 21, 22, 23	0.40	1189	19.88	1.67	0.017

The reinforcing action of the steel cords was significant for both the reinforcing schemes. As can be noted in Table 4, the average increase in strength was approximately 124% in the case of continuous wrap (Series O-3X2-C) and 67% for discontinuous wrap (Series O-3X2-D).

All specimens failed by the rupture of the SRP jacket due to hoop tension after the masonry had disintegrated (Figure 7b). Typical failure of wrapped specimens was very noisy followed by an “explosive” fracture of the steel cords. The failure was preceded by a visible transversal deformation of the specimen in about the middle. This is the most common mode of failure for FRP-confined masonry, although premature failure due to the separation of the FRP at the vertical lap joint has also been reported for specimens with an insufficient lap length.

**Figure 8:**
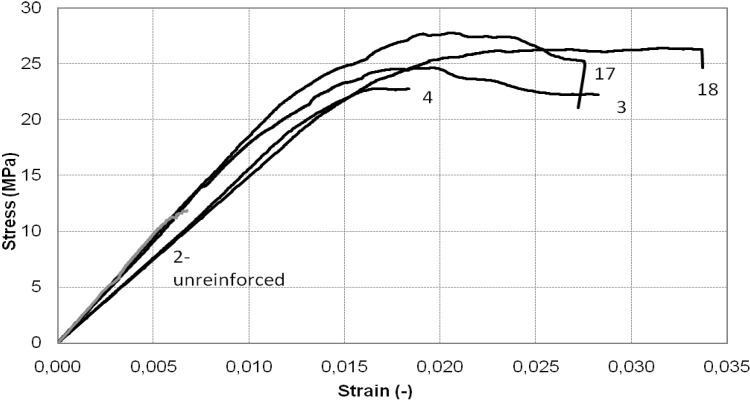
Stress-strain diagram of square cross-section column: continuous wrap.

**Figure 9:**
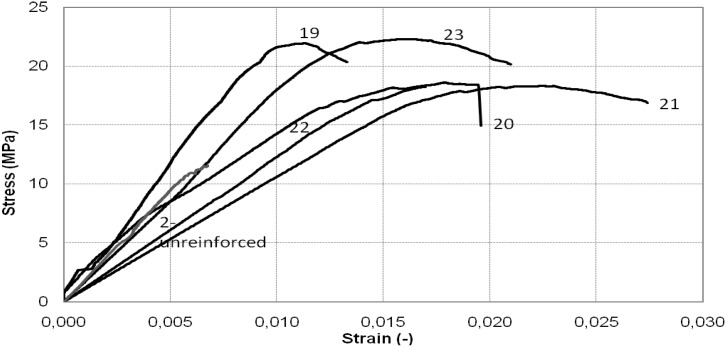
Stress-strain diagram of square cross-section column: discontinuous wrap.

Figure 8 and Figure 9 illustrate a comparison between the stress-strain curves obtained from the experimental tests on the square cross-section column. It can be observed that in all cases the columns behaved almost linearly until they reached the maximum load. At this stage, the failure mechanism started to occur and a sudden drop in the stress-strain curves was recorded.

### 4.2. Octagonal Cross-Section Masonry Specimens

Thirteen octagonal cross-section columns were tested under uniaxial compression until failure. Two test variables were considered: the type of steel cords (Type 1 and Type 2) and the configurations of the reinforcing system (continuous and discontinuous wrap). Before wrapping the SRP sheets, masonry surface defects were filled with epoxy putty. A layer of epoxy resin was next applied on the surface of each specimen, and then wrapping of the steel cords was applied with the fibers in the hoop direction. Considering the octagonal cross section of the masonry specimens, it was not necessary to use cold-bended steel cords.

**Figure 10:**
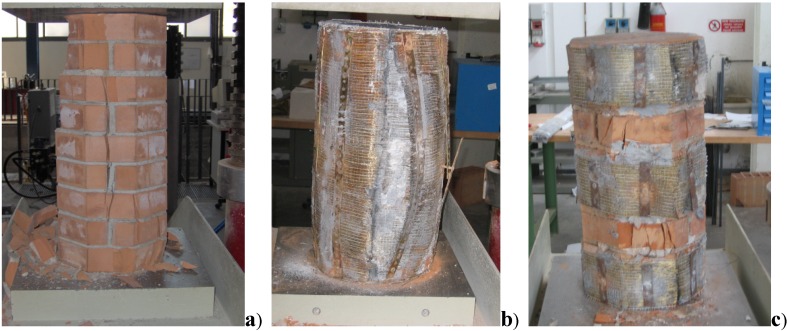
Failure modes of octagonal cross-section column: **(a)** unreinforced specimens; **(b)** reinforced specimen (continuous wrap); **(c)** reinforced specimens (discontinuous wrap).

The control specimen failed at a load of 719 kN, corresponding to a compressive strength of 14.0 MPa by the formation of vertical cracks through the head joints and the bricks. The specimen (Figure 10a) developed vertical cracks along the surfaces for an initial cracking load between 420 and 600 kN. These cracks initiated on the external surface of the masonry column and then spread inwards. Despite the fact that material properties were quite similar, the specimen with octagonal cross section was stronger.

The most important experimental result for the thirteen specimens regards the increase in strength and deformation capacity attainable in a column confined with steel cords. The results obtained showed a significant increase in strength which was more evident in the case of 3SX cords. As can be noted in Table 5 the average increase in strength was approximately 90% in the case of continuous wrap (Series O-3SX-C) and 80% for discontinuous wrap (Series O-3SX-D). Conversely, the columns confined with 3X2 cords evidenced an average increase in strength of 79% and 71% for Series O-3X2-C and O-3X2-D respectively.

With regard to the failure mode among the confined columns a strong resemblance in the failure mode was also noted (Figure 10b,c): during the initial phase of the compression tests, visible deformations were not encountered in all twelve reinforced samples: increasing the load vertical cracks formed though mortar joints and bricks and a progressive transversal dilation of the masonry columns was noted which terminated in the failure of the SRP material. At the end of the test, the confined portion of the abovementioned samples turned out to be crushed and disintegrated, and in any case more damaged compared to the less confined portions adjacent to the reinforcement, which broke off from the column and remained attached to the reinforcement with a more or less constant thickness. Failure of the sheet occurred midway up the column and involved an area covering approximately one third of column the height.

**Table 5:** Octagonal cross-section masonry columns: Experimental results.

Series	Specimen	Volumetric ratios λ (%)	Average max compression load P (kN)	Average compressive strength (MPa)	Normalized strength P_confined_/P_unconfined_	Average load peak axial strain
Unconfined	1	-	719	14.0	1.00	0.0085
O-3SX-C	5, 7, 16	0.47	1361	26.6	1.90	0.0166
O-3SX-D	6, 8, 12, 15	0.31	1293	25.2	1.80	0.0128
O-3X2-C	9, 11, 14	0.62	1289	25.2	1.79	0.0117
O-3X2-D	10,13	0.41	1224	23.9	1.71	0.0127

**Figure 11:**
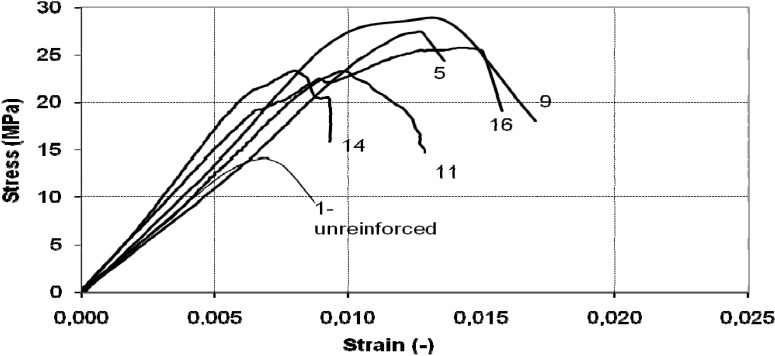
Stress-strain diagram of octagonal cross-section column: continuous wrap.

**Figure 12:**
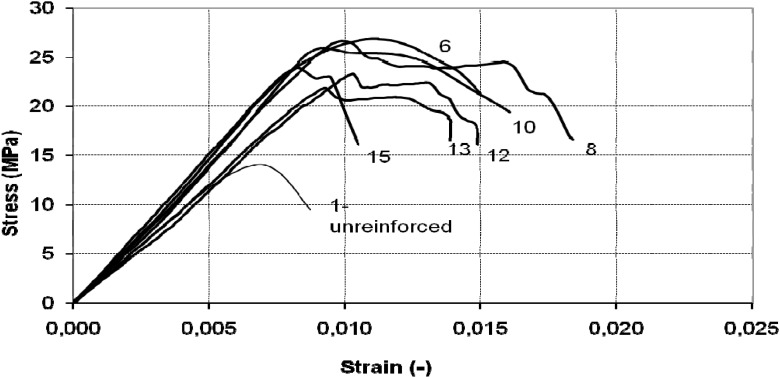
Stress-strain diagram of octagonal cross-section column: discontinuous wrap.

A significant increase in deformation capacity was also observed in all cases (Figure 11 and Figure 12). The post-peak behavior in the majority of tests was marked by abrupt loss of strength over a very narrow strain increment, accompanied by rupture of the SRP jacket.

## 5. Design

The equations suggested by Italian CNR [12] for design of FRP reinforcement, based on European Code for design of masonry structures [13] on compressive strength testing of confined masonry columns, has shown that the design compressive strength (*f_mcd_*) for members confined with FRP subjected to a lateral confining pressure (*f_1_*) can be written as follows:

(1) $$f_{mcd}=f_{md}+k\prime \cdot f_{1}\prime$$

where *f_md_* represents the design compressive strength of unconfined masonry, *k’* is a non-dimensional coefficient and *f_1_’* is the effective lateral confing pressure. The coefficient *k’* can assume different values, according to the material and the typology of the applied reinforcement. For FRP reinforcement, the value of *k’* is indicated as *g_m_*/1250 where *g_m_* is the specific weight of masonry expressed in kg/m^3^.

**Figure 13:**
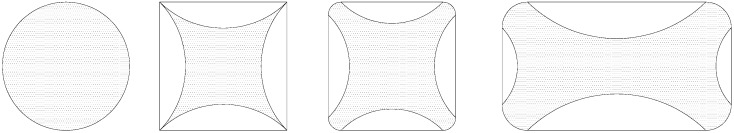
Effective areas of confinement for different cross sections.

**Figure 14:**
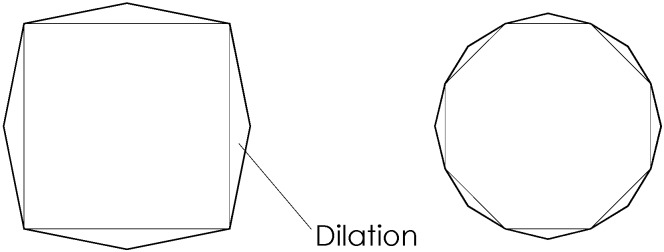
Idealization of dilated cross-sections.

The effective pressure *f_1_’* is expressed in the Standard as:

(2) $$f_{1}\prime =k_{eff}\cdot f_{1}=k_{H}k_{V}f_{1}$$

where *k_eff_* is the effectiveness coefficient. This value is the product between two terms: *k_H_* and *k_V_* related to the horizontal and vertical effectiveness. The effectiveness coefficient *k_eff_* depends on the values of the effectively confined volume *V_c,eff_* to the total volume *V_m_*. The horizontal coefficient *k_h_* takes into consideration the percentage of confined area of a wrapped section (Figure 13 and Figure 14), while the vertical effectiveness coefficient *k_V_* covers the effect of discontinuous wrapping throughout the column axis.

**Figure 15:**
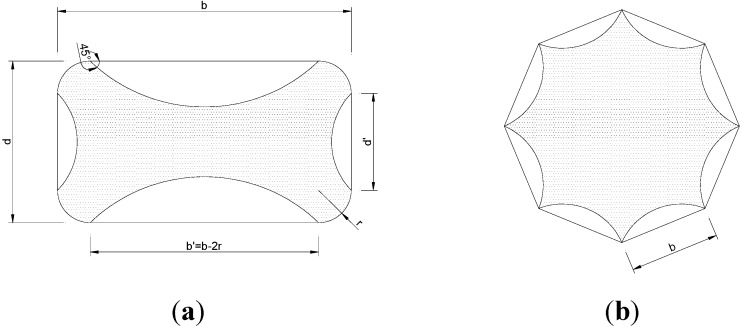
Calculation of the confined cross-section: **(a)** square and **(b)** octagonal cross-sections.

For the square cross section columns, the horizontal effectiveness coefficient is equal to:

(3) $$k_{H}=1-\frac{b\prime^{2}+d\prime^{2}}{3A_{m}}$$

where *b’* and *d’* are indicated in Figure 15a and *A_m_* is the total (gross) cross sectional area of the wrapped member.

For continuous wrapping *k_V_* =1, whereas for discontinuous wrap, the vertical effectiveness coefficient is equal to:

(4) $$k_{V}={\left(1-\frac{p_{f}+b_{f}}{2\text{min}\left(b,d\right)}\right)}^{2}$$

The nominal lateral pressure can be calculated as a function of the reduced FRP design strain *ε_fb,rid_*:

(5) $$\begin{array}{l} \varepsilon_{fb,rid}=\eta_{a}\cdot \varepsilon_{fk}/\gamma_{f}\\ f_{1}=\frac{1}{2}\left(\rho_{f}\cdot E_{f}\right)\varepsilon_{fb,rid} \end{array}$$

where *η_a_* and *γ_f_* represent environmental conversion factor and partial factor as suggested by CNR code; *ε_fk_* is the characteristic axial strain of composite; *E_f_* is the Young modulus of the SRP sheet; *ρ_f_* is the wrapping ratio depending on the thickness of the composite layer *t_f_*:

(6) $$\rho_{f}=\frac{2t_{f}b_{f}}{bp_{f}}$$

where *b_f_* is the width of the wrapping along the vertical direction (fibers are supposed to be at 90° with respect to the principal axis of the masonry column), *b* is the highest dimension of the cross-section and *p_f_* is the spacing length between two consecutive SRP sheets measured vertically.

The design value of the SRP-confined strength, according to equation (1) provided by the mentioned Italian Guidelines for FRP reinforcement [12], is 1400 kN and 952 kN for continuous and discontinuous wrap (Type 2 cord) respectively, which is in good accordance with the experimental value (Table 6).

**Table 6:** Comparison between experimental and theoretical values.

Series	*k_eff_*	*f_1_*	*f’_1_*	Maximum compression load P_theoretical_ (kN)	Maximum compression load P_experimental_ (kN)	P_experimental_ /P_theoretical_
**S-3X2-C**	0.497	13.95	6.93	1400	1510	1.07
**S-3X2-D**	0.315	7.11	2.83	952	1189	1.24

## 6. Conclusions

Problems in upgrading existing masonry structures to conform to current anti-seismic specifications are often encountered by engineers and other technicians. In some cases problems can be resolved through the use of SRP materials which present certain advantages compared to the traditional reinforcing technique.

Experiments were carried out on twenty-three masonry columns having two different cross-sections, square and octagonal. Octagonal cross-section masonry columns are common in many historical constructions and no data are present in bibliography on their mechanical behavior.

For all columns, reinforcement consisted of one layer of Type 1 or Type 2 steel cords applied as external reinforcement along the perimeter of the masonry columns in continuous discontinuous wrap forms. The increase in strength and deformation capacity measured for these columns was significant and SRP wrapping demonstrated greater effectiveness. The greater efficacy of the reinforcement was highlighted particularly in those cases in which Type 1 cords were used. In each case, the failure mode of confined columns were similar: at the end of the test, the confined portion of the samples was crushed and disintegrated, and certainly was more damaged than the less confined portions adjacent to the reinforcement, which broke off from the column and remained attached to the reinforcement with a more or less constant thickness. Failure of the sheet occurred midway up the column and involved an area covering approximately one third of the column height.

A comparison of experimental results and the predictions obtained by analytical formulations (Equations (1) to (6)) was made to evaluate the possibility of using such formulations to predict the behavior of the strengthened columns. The analytical formulations presented in this paper could represent a first step for the development of code recommendations for the design of strengthening of masonry columns using steel cords. This comparison reveals good agreement between the experimental data and theoretical predictions for the corresponding load-carrying capacity
